# Doxycycline in Hospitalized Patients with Dengue

**DOI:** 10.3390/tropicalmed11100277

**Published:** 2026-09-30

**Authors:** Roberto Alfonso Accinelli, Cesar Eduardo Malca-Polo, Kevin Jesus Salas-Espinoza, Wilbert Jorgam Ramos-Mamani

**Affiliations:** 1 Instituto de Investigaciones de la Altura, Universidad Peruana Cayetano Heredia, Lima 15102, Peru; kevin.salas.e@upch.pe (K.J.S.-E.); wilbert.ramos.m@upch.pe (W.J.R.-M.); 2 Facultad de Medicina Alberto Hurtado, Universidad Peruana Cayetano Heredia, Lima 15102, Peru; 3 Hospital Cayetano Heredia, Lima 15102, Peru; 4 Clínica Fátima, Trujillo 13009, Peru; dr.cesarmalca@gmail.com

**Keywords:** dengue, doxycycline, thrombocytopenia, hospitalization, symptoms

## Abstract

Objective: The objective of this study was to determine whether individuals with dengue who received doxycycline had a shorter hospitalization time. Methods: An observational, retrospective, and comparative study was conducted in an anonymized database with 64 hospitalized dengue patients from Clinic Fatima, Trujillo, Peru. They were diagnosed and treated according to the Peruvian Ministry of Health guidelines. Of the patients, 47 received doxycycline with an initial dose of 200 mg, followed by 100 mg every 12 h until discharge. The remaining 17 received only symptomatic treatment in accordance with Peruvian guidelines. Clinical and laboratory variables were compared between groups. Results: Both groups had similar ages, sex distribution and pre-hospitalization fever time, with dengue being the only infectious disease that they had. The doxycycline group had significantly shorter pre-hospitalization durations of headache (103.15 ± 26.30 vs. 125.68 ± 31.21 h, *p* = 0.009) and longer myalgia (97.02 ± 19.35 vs. 81.88 ± 19.08, *p* = 0.014) time. Compared to patients who received only the standard treatment, those treated with doxycycline had a shorter hospitalization time (26.30 ± 13.72 vs. 93.18 ± 25.29 h, *p* < 0.0001), as well as shorter durations of fever (13.79 ± 16.18 vs. 127.08 ± 51.22, *p* < 0.0001), headache (16.30 ± 19.27 vs. 94.59 ± 26.11, *p* < 0.0001), and myalgia (23.94 ± 10.90 vs. 120.24 ± 25.20, *p* < 0.0001). Furthermore, the doxycycline group exhibited a higher platelet recovery rate (0.54 ± 0.49 vs. 0.23 ± 0.29 × 10^3^/µL·h^−1^, *p* = 0.003), and none of them discontinued it due to adverse reactions. Despite limitations, including the sample size and lack of randomization, the findings suggest that doxycycline may represent a promising and useful therapeutic alternative in the management of dengue. Conclusions: The use of doxycycline in dengue treatment was associated with reduced hospitalization time, shorter symptom duration, and a higher platelet recovery rate.

## 1. Introduction

Dengue is an infectious disease caused by the dengue virus (DENV). This systemic viral infection, colloquially termed *break-bone fever*, is common in tropical and subtropical areas, where 75% of symptomatic cases occur each year [1]. The principal vectors responsible for transmission are *Aedes aegypti* and *Aedes albopictus* mosquitoes [2]. Notably, *A. albopictus* was absent in South America until recent years, but due to global mobility and climate change, it has now become established in the region [3]. Consequently, the incidence of dengue has risen substantially, even in temperate cities such as Buenos Aires, where reported cases increased from 14 in 2015 [4] to 55,375 in 2024 [5]. Similarly, Peru reported 262,287 cases and 252 deaths in the same year [6]. Globally, cases reported to the World Health Organization (WHO) have increased dramatically, from 505,430 in 2000 to 5.2 million in 2019, reaching more than 14.3 million in 2024 [1].

Previous data suggest that only one in four individuals infected for the first time develops symptoms [3]. Therefore, the true incidence of dengue is substantially underestimated due to the high frequency of subclinical and mild self-limiting infections. More than 90% of cases are mild, with a fatality rate of about one in every 1000 to 2000 cases, but when it becomes severe dengue, the fatality rate increases to between 1% and 5% [7]. The symptomatic phase typically follows an incubation period of 4–10 days and generally lasts for 2–7 days. Characteristic clinical presentation often includes high-grade fever, severe cephalgia, retro-orbital pain, myalgia, arthralgia, and variable cutaneous manifestations [1]. Dysregulation of the immune responses is considered a key mechanism by which DENV promotes survival and induces severe disease manifestations in the host [8]. The increased clinical severity and disease progression of dengue have been associated with excessive inflammation, with raised IL-6, CXCL10, TNF-α [9] and other inflammatory cytokine levels [8].

Management remains exclusively supportive, with non-steroidal anti-inflammatory drugs (NSAIDs) contraindicated [1,10]. Current treatment strategy focuses on adequate volume replacement and, in patients with bleeding manifestations, the administration of blood products [11]. CYD-TDV is a live-attenuated tetravalent chimeric vaccine [3] recommended for individuals aged 9 to 45 with a history of prior infection [12]. TAK-003 is a recombinant live-attenuated chimeric vaccine [3] that reduces hospitalizations by 84% and symptomatic cases by 61% [13], with current indication restricted to children and adolescents aged 6–16 years residing in high-transmission endemic areas [1].

Although dengue has a relatively low case fatality rate, it remains the most prevalent arthropod-borne viral disease worldwide [14]. Current epidemiological estimates indicate that approximately half of the global population resides in regions at risk of DENV infection [1]. This substantial disease burden, combined with the limitations of current prophylactic options, highlights the urgent need for specific antiviral therapies [15]. Several drugs have, therefore, been investigated for repurposing against dengue [16], though most have demonstrated negligible impact on clinical outcomes. One notable exception is doxycycline, a second-generation tetracycline-class antibiotic. Beyond its established antibacterial mechanism of inhibiting protein synthesis via binding to the 30S ribosomal subunit, evidence suggests it may provide beneficial clinical effects in dengue. Previous studies report that patients receiving doxycycline experienced a faster resolution of symptoms, shorter hospitalization, improved recovery from thrombocytopenia and leukopenia, and reduced mortality [17,18]. These effects may be explained by multiple mechanisms. Doxycycline appears to inhibit DENV replication by preventing viral entry through binding to a key hydrophobic pocket measuring 481 Å in the viral envelope (E) protein [10,19], thereby sterically hindering the conformational changes required for viral entry [10,20,21]. In addition, doxycycline inhibits intracellular viral replication by targeting the DENV serine protease NS2B-NS3 complex [19]. Furthermore, it exerts immunomodulatory effects by reducing levels of pro-inflammatory cytokines such as TNF-α and IL-6 [18,22].

The most frequently reported adverse reactions to doxycycline involve the gastrointestinal system, including heartburn, nausea, vomiting, diarrhea, and gastritis. Reported frequencies vary widely, ranging from 0.54% to 51.7% [23], with one study reporting that adverse reactions occur in 15.7% of patients [24]. Unlike other tetracyclines, doxycycline has not been associated with tooth staining, enamel hypoplasia, or tooth discoloration in children who received short-term treatment before the age of 8 years [25].

Therefore, this study was designed to contribute to the growing body of evidence regarding doxycycline use in dengue by evaluating clinical and hematological outcomes, particularly platelet count dynamics, through a retrospective analysis of a pre-existing, anonymized patient database. The dataset included individuals hospitalized for dengue at Fatima Clinic, a private hospital in Trujillo, Peru (34 m above sea level). Therapeutic decisions had originally been made at the discretion of their attending physicians, which resulted in two cohorts for comparative analysis: one treated with the internationally recommended standard supportive care alone, and another that received adjunctive oral doxycycline on a compassionate-use basis.

## 2. Methods

An observational, retrospective, and comparative study was designed using a cohort of hospitalized dengue patients treated at Fatima Clinic, a private hospital in Trujillo, Peru, during 2024. Patients presenting to the Emergency Department with fever, epidemiological exposure and other clinical criteria consistent with dengue according to the guidelines of the Peruvian Ministry of Health (MINSA in Spanish, Ministerio de Salud) and who also met hospitalization criteria were included in this study (Supplementary Table S1) [26]. Upon admission, all patients received the standard therapeutic management according to current MINSA regulations (Supplementary Table S1). Additionally, on a compassionate-use basis, attending physicians could propose adjunctive treatment with doxycycline to patients. Treatment was administered to those who accepted. This real-world, non-randomized allocation resulted in the formation of two cohorts for comparison: a control cohort (*n* = 17), consisting of patients managed exclusively with standard supportive care, and a doxycycline cohort (*n* = 47). The anonymized database provided for analysis contained no personally identifiable information.

For all patients, data regarding age, sex, and duration (in hours) of headache, fever and myalgias prior to admission were recorded. Blood tests were performed at admission and throughout hospitalization. Also, NS1 antigen and IgM serology were requested; however, diagnosis and treatment decisions were not contingent upon positive results [26].

The primary objective of this study was to determine whether individuals who received doxycycline experienced a shorter hospitalization time compared to those managed exclusively according to MINSA guidelines. Secondary objectives included evaluating symptom duration and platelet count dynamics. For each patient, the platelet recovery rate was calculated as a measure of hematologic recovery velocity. This metric was defined as the difference between platelet counts at discharge and admission divided by hospitalization time, providing a standardized estimate of platelet recovery dynamics (expressed as ×10^3^/µL·h). No other laboratory data were in the anonymized database.

The normality of continuous variables was assessed using the Shapiro–Wilk test. For variables with a normal distribution (*p* > 0.05), between-group comparisons were performed using the independent Student’s *t*-test, with results reported as mean differences with 95% confidence intervals (CIs). For continuous variables that violated the assumption of normality, the non-parametric Mann–Whitney U test was used; these data are presented as median and interquartile range (IQR). Normally distributed variables are presented as mean ± standard deviation (SD). Table 1 includes both descriptive measures to provide a comprehensive characterization of the data distribution and ensure consistency in interpretation. Effect sizes were calculated using Hedges’ g.

Statistical analyses and graphical visualizations were performed using Python (version 3.11.10) with the Pandas (version 2.0.3), NumPy (version 1.24.3), Seaborn (version 0.12.2), and Matplotlib (version 3.7.1) libraries. Data distributions were visualized using violin plots generated via kernel density estimation. These plots included an embedded boxplot depicting the median and interquartile range, with overlaid individual data points.

Ethical approval for this retrospective study, which used a fully anonymized patient database, was granted by the Institutional Ethics Committee of the Universidad Peruana Cayetano Heredia (Approval Number: 217526, dated 14 April 2025). The requirement for informed consent was waived by the committee due to the retrospective nature of the study and the exclusive use of anonymized data.

## 3. Results

This study included 64 patients hospitalized with dengue, comprising 31 women and 33 men, with a mean age of 30.8 years. All received standard symptomatic care per MINSA guidelines, but 47 also received doxycycline (an initial dose of 200 mg, followed by 100 mg every 12 h until discharge). There were 22 women and 25 men in the doxycycline group, and 9 women and 8 men in the other (*p* = 0.48). Because they were young people, in the doxycycline group, there were only five patients older than 48 years, with two of them having diabetes mellitus type 2 and one arterial hypertension, and in the rest of the group, there was another patient with obesity. Among the 17 who did not receive doxycycline, nobody had comorbidities, with two 48-year-old patients being the oldest.

All patients presented with thrombocytopenia and symptoms of fever, headache, and myalgia upon admission. There were no differences between groups in terms of age, hours of fever before hospitalization or sex distribution. However, the doxycycline group had significantly shorter pre-hospitalization headache (mean difference: −22.53 h; 95% CI: −44.12 to −0.94; g = −0.95) and longer myalgia (mean difference: 15.14 h; 95% CI: 4.51 to 25.77; g = 0.78) times, with moderate-to-large effect sizes (Table 1 and Figure 1A–C).

Compared to the standard care group, patients treated with doxycycline experienced markedly better outcomes during hospitalization. They had a significantly shorter hospitalization time (26.30 ± 13.72 vs. 93.18 ± 25.29 h, *p* < 0.0001; Δ = −66.88 h; 95% CI: −82.29 to −51.47; g = −4.29), as well as shorter durations of fever (13.79 ± 16.18 vs. 127.08 ± 51.22 h, *p* < 0.0001; Δ = −113.29 h; 95% CI: −140.52 to −86.06; g = −3.81), headache (16.30 ± 19.27 vs. 94.59 ± 26.11 h, *p* < 0.0001; Δ = −78.29 h; 95% CI: −98.64 to −57.94; g = −3.36), and myalgia (23.94 ± 10.90 vs. 120.24 ± 25.20 h, *p* < 0.0001; Δ = −96.30 h; 95% CI: −109.55 to −83.05; g = −5.99). Furthermore, the doxycycline group exhibited a significantly higher platelet recovery rate (0.54 ± 0.49 vs. 0.23 ± 0.29 ×10^3^/µL·h^−1^, *p* = 0.003; Δ = +0.31 ×10^3^/µL·h^−1^; 95% CI: +0.14 to +0.48; g = +0.75) (Table 1 and Figure 2). No adverse effects related to the doxycycline treatment were reported. No patient had another infectious disease simultaneously with dengue. This analysis is based on data collected until the moment of discharge, with no follow-up information available thereafter.

## 4. Discussion

The use of doxycycline in dengue treatment was associated with a significant reduction in hospitalization time and the duration of major symptoms and an increased platelet recovery rate. Regarding symptoms prior to hospitalization, the duration of fever, the longest-lasting symptom before admission, was comparable between groups. However, baseline imbalances were observed for headache and myalgia duration, which were 22.53 h shorter and 15.14 h longer, respectively, in the doxycycline group (Table 1 and Figure 1). Despite these differences, the treatment effect during hospitalization was substantially larger. Doxycycline treatment was associated with a reduction of 66.88 h in hospitalization time, 78.2 h in headache, 96.3 h in myalgia and 113.29 h in fever duration. (Table 1 and Figure 2). This considerable magnitude of these improvements supports the interpretation that the observed clinical benefits were primarily associated with the therapeutic intervention.

Furthermore, although the platelet counts at admission were similar between groups, hematological recovery appeared to be accelerated in patients receiving doxycycline. The platelet recovery rate was more than twofold higher in the doxycycline group compared to the control group (0.54 ± 0.49 vs. 0.23 ± 0.29 × 10^3^/µL·h^−1^, *p* = 0.003) (Table 1 and Figure 2).

Fredeking et al. [18] reported that among patients with hemorrhagic dengue and plasma leak, those randomly assigned to receive oral doxycycline plus WHO-recommended therapy (*n* = 116) had a 46% lower mortality compared to patients receiving standard therapy alone (*n* = 115) (11.2% [13/116] vs. 20.9% [24/115], *p* = 0.05). In addition, the doxycycline group showed a significant reduction in TNF and IL-6 levels (*p* < 0.01) during follow-up [18]. Similarly, Pambhar et al. [17] conducted a clinical study with 120 thrombocytopenic dengue patients. Patients without leukopenia (*n* = 65) received standard WHO therapy, whereas patients with leukopenia (*n* = 55) were stratified by platelet count. Those with platelet counts between 50,001 and 150,000/μL received doxycycline, and those with counts below 50,001/μL received doxycycline combined with *Carica papaya* leaf extract. The study demonstrated improvements in both platelet and leukocyte counts on days 4 and 7, with a graded therapeutic effect observed among groups: WHO therapy < doxycycline < doxycycline with *Carica papaya*. Consistently, the mean duration of hospitalization followed the same pattern, being the longest in the control group (9.1 days), intermediate in the doxycycline-only group (8.2 days), and the shortest in the combination therapy group (7.3 days) [17].

Through virtual screening (VS) using the Generic Evolutionary Method for Molecular Docking (GEMDOCK) applied to the Comprehensive Medicinal Chemistry (CMC) database, doxycycline was among the top 10 leading candidates of the 5331 screened molecules. Candidate compounds were selected based on structural similarities, docked positions, protein–ligand interactions and commercial availability limitations. Experimentally, doxycycline at 500 µM reduced plaque-forming units by 99%, exhibiting a median inhibitory concentration (IC_50_) of 55.6 µM. Computational modeling has suggested that the binding capacity of tetracycline-class molecules may be partially explained by structural similarities to the bacterial TetR protein, whose binding site volume (359–495 Å) is comparable to the dengue viral E protein itself. When doxycycline occupies this pocket, its side chains form hydrogen bonds with residues on opposite walls, generating steric hindrance that interferes with the conformational changes required for viral entry. The resulting doxycycline-bound complex is more stable than the analogous complex formed with tetracycline [10].

Doxycycline directly inhibits the DENV2 NS2B-NS3 protease (NS2B-NS3pro), with an IC_50_ value of 52.3 ± 6.2 µM at 37 °C and 26.7 ± 5.3 µM at 40 °C indicating enhanced inhibitory activity at febrile temperatures. This mechanistic finding is supported by cell culture evidence, where treatment with 50 µM doxycycline for 72 h significantly reduced viral copies across all serotypes, with a more pronounced effect observed against DENV2 and DENV4 compared to DENV1 and DENV3 [19].

The superior binding affinity of doxycycline compared to tetracycline, characterized by more stable and numerous hydrogen bonds with the pocket walls [10], may help explain its enhanced clinical efficacy. This structural advantage has also been reflected in clinical studies where doxycycline demonstrated more effective immunomodulatory activity. Castro et al. [22] reported that 45 dengue patients treated with doxycycline exhibited a more favorable modulation of elevated cytokine levels and cytokine receptors/antagonists compared to 35 patients receiving tetracycline. By contrast, among 34 untreated patients, IL-6, IL-1β, and TNF levels remained elevated or increased further over time. Both tetracycline groups demonstrated reductions in these inflammatory markers. Furthermore, by days 3 and 7, IL-1RA and TNF-R1 levels had increased significantly from baseline in all three study groups [22].

The beneficial antiviral effects of doxycycline have also been documented in other viral diseases. In Vero cells infected with chikungunya virus (CHIKV), doxycycline inhibited viral replication, with an EC_50_ of 10.9 μM, which is lower than that of 15.5 μM of ribavirin alone, though higher than the EC_50_ achieved with the doxycycline–ribavirin combination (4.5 μM). In vivo experiments showed that treatment with doxycycline–ribavirin led to a significant reduction in virus titer in the blood of ICR mice at 7 days post-infection. Docking studies with the CHIKV nsP2 cysteine protease and E2 glycoprotein indicated that the compound could bind to both of these viral targets. However, cell-based assays indicated that the principal antiviral mechanism likely involves the inhibition of viral entry through interference with conformational changes in the E2 glycoprotein. CHIKV particles were also inactivated after one hour of incubation with doxycycline, which suggests a possible direct interaction with the viral envelope [27].

Similarly, doxycycline has demonstrated antiviral activity against Zika virus. The binding of doxycycline to the ZIKA NS2B-NS3 complex is generated by four hydrogen bonds with the protease residues TYR 1130, SER 1135, GLY1151, and ASP83. In silico analyses showed that doxycycline exhibited a stronger binding affinity to the ZIKV NS2B-NS3 compared to the standard boronate inhibitor. Notably, doxycycline displayed a greater inhibitory effect at 40 °C (IC_50_ = 5.3 µM) than at 37 °C (9.9 µM). Increasing doxycycline concentrations also reduced the maximum reaction velocity. Time-of-addition assay demonstrated that doxycycline significantly inhibited virus entry, although its effect on virus replication appeared more efficient [28].

Antiviral activity has additionally been reported against Japanese encephalitis virus (JEV). In infected cell cultures, doxycycline reduced viral RNA replication in a dose-dependent manner. These in vitro findings are also supported by in silico studies showing favorable interactions between doxycycline and motifs I, II and VI of JEV NS3 helicase/NTP, which suggests potential inhibition of viral replication mechanisms [29]. In adult Swiss albino mice infected with JEV, doxycycline treatment at a 50 mg/kg dose initiated reduced disease progression 24 h post-infection, prolonged survival by approximately one week, and lowered viral titers. Viral titration of JEV on harvested brain tissue from deceased mice also demonstrated a marked reduction in plaque formation [30].

Mosquitoes of the genus Aedes are the principal transmitters of vector-borne diseases, including chikungunya, Zika infection, and dengue. Japanese encephalitis virus (JEV), by contrast, is transmitted primarily by mosquitoes of the genus Culex. All of them are positive-sense, single-stranded RNA viruses of the genus *Flavivirus*, with the exception of chikungunya, which is an *Alphavirus.* Doxycycline may represent an effective treatment for these infectious agents because *Flavivirus* has similar places in its structure, including the envelope E protein and the NS2B-NS3 complex, which constitute proposed targets for doxycycline-mediated inhibition of viral entry and intracellular replication.

Several repurposed drugs have been evaluated for dengue treatment, with limited success. Chloroquine showed in vitro activity against DENV [31] and provided symptomatic relief by reducing pain in patients, but it did not significantly affect disease duration, symptom intensity, or fever duration in clinical trials [32]. The conflicting evidence regarding its efficacy, with some studies showing no therapeutic benefit [31], highlights the challenges of translating in vitro findings to clinical practice. Interestingly, chloroquine demonstrated prophylactic potential by preventing the development of specific IgG antibodies in infected monkeys [33]. Similarly, ivermectin, which inhibits importin alpha/beta-mediated nuclear import [34], suppresses DENV replication across all four serotypes in vitro. While clinical evidence remains limited, studies have reported effects on NS1 antigen clearance and reduced NS1 persistence at hospital discharge [35,36], suggesting potential antiviral activity that merits further evaluation in rigorous clinical trials.

The safety profile of doxycycline is favorable [37]. In a population-based study in Iceland, it was estimated that 16,339 patients would need to be treated with doxycycline to observe a single case of drug-induced liver injury (DILI) [38]. Furthermore, a California Medicaid claims database study found no increased risk of DILI among current or past users of doxycycline compared with non-users [39].

This study has several important limitations. First, baseline imbalances in pre-hospitalization headache and myalgia duration may have contributed to an overestimation of the apparent treatment effect. Second, the sample size was modest (*n* = 64). Third, non-randomized treatment assignment resulted in unequal group sizes (47 vs. 17). Fourth, doxycycline was administered on a compassionate-use basis according to the discretion of attending physicians, which may have introduced selection bias if clinicians preferentially offered treatment to patients perceived as having a better prognosis.

Although randomized controlled trials (RCTs) are commonly regarded as the higher standard for establishing therapeutic efficacy, Concato et al. [40] demonstrated that well-designed observational studies can yield results comparable to RCTs, sometimes with lower heterogeneity in effect estimates. Their analysis suggested that observational studies do not necessarily overestimate treatment adverse effects and may provide a broader representation of the at-risk population [40]. Nevertheless, consistent with conservative scientific principles, findings derived from observational studies such as the present investigation should primarily be considered hypothesis-generating [41]. Consequently, despite the promising results observed, these findings should not prompt immediate modifications in clinical practice until confirmed through adequately powered randomized controlled trials, as premature implementation could potentially lead to unintended adverse outcomes [41].

The consistency of our findings with previous clinical [17,18] and in vitro investigation [31], together with the existence of a plausible stereochemical mechanism of antiviral action, provides a strong rationale for future rigorous clinical trials with doxycycline, not only in dengue but also in *Flavivirus* and *Alphavirus* infections, whose structural characteristics may permit the inhibition of viral entry and replication by doxycycline.

## 5. Conclusions

In conclusion, the use of doxycycline was associated with a significantly shorter hospitalization time, reduced symptom duration, and faster recovery from thrombocytopenia in this cohort of hospitalized dengue patients from coastal Peru.

## Figures and Tables

**Figure 1:**
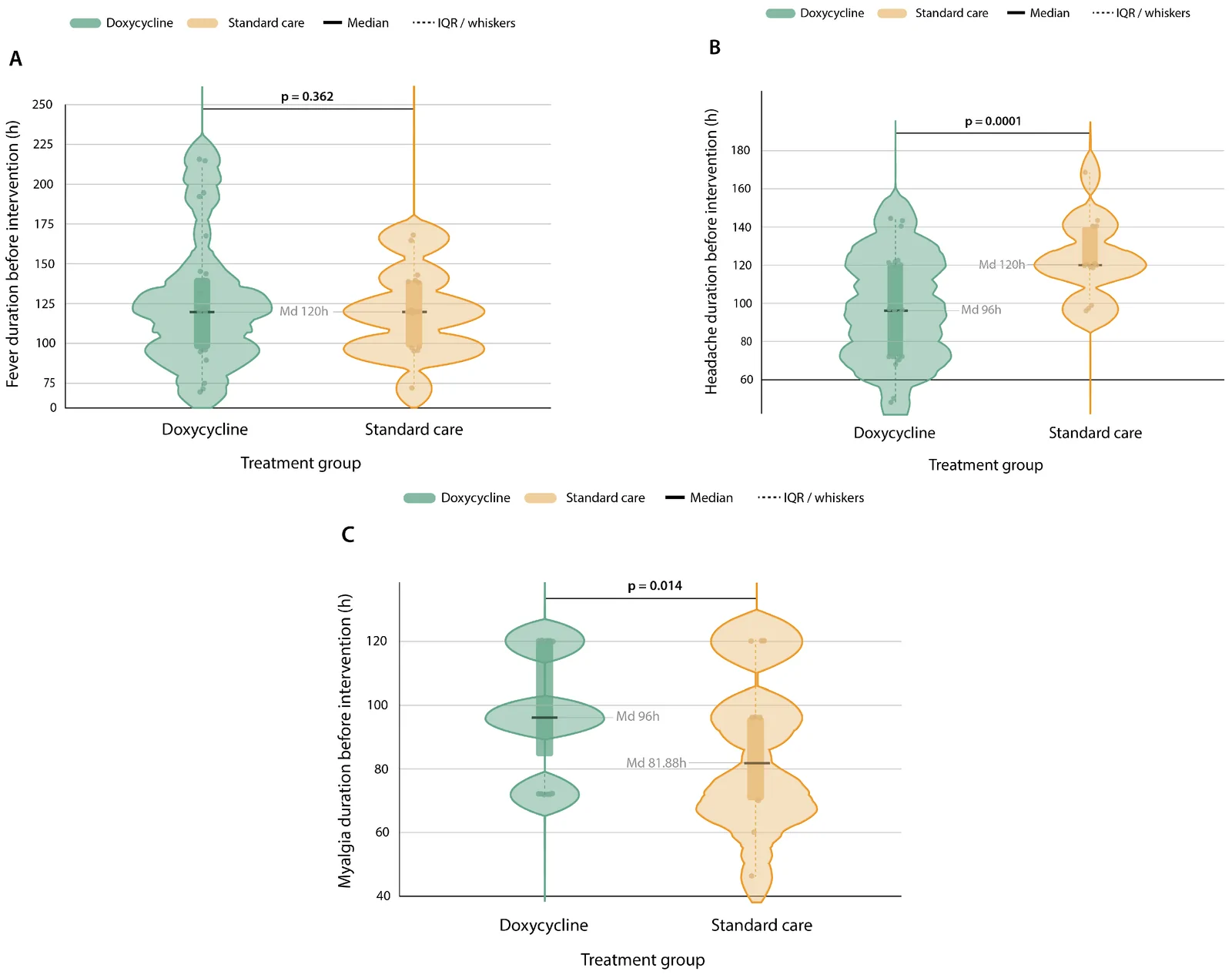
Distribution of pre-hospitalization symptom duration in hospitalized dengue patients according to treatment group (doxycycline vs. standard symptomatic care). (**A**) Fever duration before admission. (**B**) Headache duration before admission. (**C**) Myalgia duration before admission. The width of the violins represents kernel density estimation. Embedded boxplots indicate the median and interquartile range, while individual data points represent single patients.

**Figure 2:**
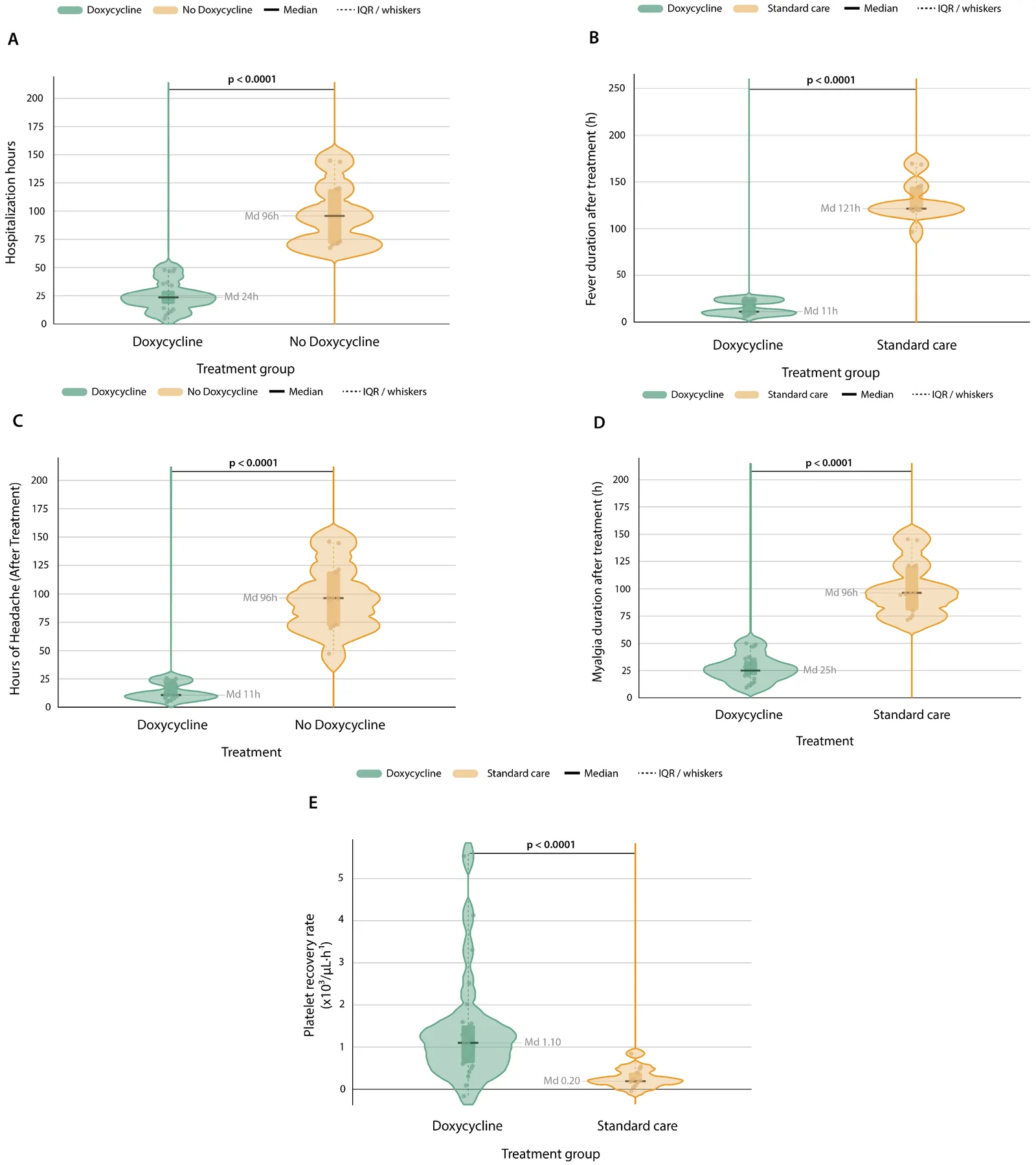
Distribution of clinical and hematological outcomes in hospitalized dengue patients according to treatment group (doxycycline vs. standard symptomatic care). (**A**) Hospitalization time. (**B**) Fever duration after intervention. (**C**) Headache duration after intervention. (**D**) Myalgia duration after intervention. (**E**) Platelet recovery rate (×10^3^/µL·h^−1^), calculated as the difference between discharge and admission platelet counts divided by hospitalization time. The width of the violins represents kernel density estimation. Embedded boxplots indicate the median and interquartile range, while individual data points represent single patients. Statistical comparisons were performed using the Mann–Whitney U test.

**Table 1:** Characteristics and Evolution of Dengue Patients. The data are presented as mean ± standard deviation (SD). Additionally, analyses of median and interquartile range (IQR) were performed, showing results consistent with those reported.

Variable	Doxycycline (*n* = 47)	No Doxycycline (*n* = 17)	*p* Value	Mean Difference (95% CI)	Hedges’ g
Age (years)	30.32 ± 11.35	32.00 ± 9.56	0.589	−1.68 (−7.86 to 4.50)	−0.15
**Pre-hospitalization**					
Fever duration (h)	114.38 ± 82.41	120.00 ± 24.00	0.362	−5.62 (−31.73 to 20.49)	−0.07
Headache duration (h)	103.15 ± 26.30	125.68 ± 31.21	0.009	−22.53 (−44.12 to −0.94)	−0.95
Myalgia duration (h)	97.02 ± 19.35	81.88 ± 19.08	0.014	15.14 (4.51 to 25.77)	0.78
**During hospitalization**					
Fever duration (h)	13.79 ± 16.18	127.08 ± 51.22	<0.0001	−113.29 (−140.52 to −86.06)	−3.81
Headache duration (h)	16.30 ± 19.27	94.59 ± 26.11	<0.0001	−78.29 (−98.64 to −57.94)	−3.36
Myalgia duration (h)	23.94 ± 10.90	120.24 ± 25.20	<0.0001	−96.30 (−109.55 to −83.05)	−5.99
Hospital stay (h)	26.30 ± 13.72	93.18 ± 25.29	<0.0001	−66.88 (−82.29 to −51.47)	−4.29
Platelet count at admission (×10^3^/µL)	68.53 ± 26.98	65.40 ± 26.60	0.307	3.13 (−11.28 to 17.54)	0.14
Platelet count at discharge (×10^3^/µL)	95.36 ± 24.22	92.28 ± 26.10	0.307	3.08 (−9.02 to 15.18)	0.15
Platelet recovery rate (×10^3^/µL·h^−1^)	0.54 ± 0.49	0.23 ± 0.29	0.003	0.31 (0.14 to 0.48)	0.75

Standard deviation (SD). Interquartile range (IQR).

## Data Availability

The data presented in this study are available on request from the corresponding author.

## Supplementary Materials

The following supporting information can be downloaded at: https://www.mdpi.com/article/10.3390/tropicalmed11100277/s1, Table S1: Data extracted from the Health Technical Standard for the Comprehensive Care of Patients with Dengue in Peru.
