# Supplementary material for: Doxycycline in Hospitalized Patients with Dengue
Source: Trop Med Infect Dis. 2026 Sep 30;11(10):277. doi: 10.3390/tropicalmed11100277 (PMC13709413; doi:10.3390/tropicalmed11100277)
Supplement: Supplementary file 1 [file tropicalmed-11-00277-s001.zip › tropicalmed-4252720-supplementary.pdf]

**Table S1: Data Extracted from Health Technical Standard for the Comprehensive Care of Patients with Dengue in Peru<br>[Supreme Decree No. 211–MINSA/DGIESP–2024, approved by Ministerial Resolution No. 175-2024-MINSA (127pp.)]**
**DEFINITIONS**<br>**Probable case of dengue without warning signs**<br>Refers to a person with fever for seven days or less, who resides in or has visited areas of dengue transmission or areas infested with the A*edes aegypti* vector within the 14 days prior to symptom onset, and who presents at least two of the following manifestations: headache, myalgia, arthralgia, ocular or retro-ocular pain, nausea/vomiting, and rash.<br>**Confirmation by epidemiological link for dengue**<br>Confirmation by epidemiological link (in outbreak or epidemic situations) is considered for any probable case that meets the case definition for dengue (epidemiological criterion plus clinical criterion) and for which, within a radius of 200 meters from the probable place of infection or residence, laboratory-confirmed cases are identified with symptom onset dates within the two weeks prior to symptom onset of the probable case to be classified.
**EVALUATION AND DIAGNOSIS**<br>The diagnosis of dengue is **FUNDAMENTALLY CLINICAL**. A patient with symptoms, signs, and epidemiological linkage should be managed as a case of dengue without requiring laboratory confirmation. Therefore, laboratory tests for the diagnosis of dengue are only diagnostic tools and do not condition the patient's treatment.
**CLASSIFICATION AND TREATMENT**<br>The key lies in the early identification and classification of cases. Every probable dengue patient should be managed comprehensively, also considering coexisting conditions such as pregnancy, comorbidities, age groups, and social risk.
The assessment of each case, and at each moment, must be highly dynamic. Health personnel at the first level of care apply a stepwise approach to perform a general evaluation, diagnosis, and correct treatment indication for dengue.<br>• **Group A (Dengue without warning signs)**: Patients who can receive home treatment under medical supervision.<br>• **Group B1 (Dengue without warning signs plus associated factors or risk)**: Patients requiring hospitalization in dengue clinical surveillance units or in a health facility with hospitalization services for monitoring and management of the infection, as well as their associated conditions or risk factors. Regarding monitoring: every patient must be observed and treated in the health facility (observation room and/or hospitalization) with clinical monitoring at least every hour.<br>• **Group B2 (Dengue with warning signs)**: Patients requiring hospitalization for intravenous fluid administration in dengue clinical surveillance units or hospitals.<br>• **Group C (Severe dengue)**: Patients requiring emergency treatment and who must be transferred and/or urgently referred to an Intensive Care Unit.
**Dengue Febrile Unit (12-hour Dengue Febrile Unit or 24-hour Dengue Clinical Surveillance Unit)**<br>• For observation of patients without warning signs who have fever and/or oral intolerance.<br>• Dengue Clinical Surveillance Unit: Hospitalization for the management of patients with dengue without warning signs but with associated factors or risk, and for patients with warning signs.<br>• Intensive Care Unit: Hospitalization for the management of patients with severe dengue.

Source: Data extracted from the Health Technical Standard for the Comprehensive Care of Patients with Dengue in Peru (127 pages; see full reference). Ministry of Health of Peru. NTS N° 211-MINSA/DGIESP-2024: Norma Técnica de Salud para la Atención Integral de Pacientes con Dengue en el Perú [Internet]. Lima: Ministry of Health; 2024 [cited 2025 Jun 11]. Available from: https://www.gob.pe/institucion/dirislimanorte/informes-publicaciones/6538529-norma-tecnica-de-salud-para-la-atencion-integral-de-pacientes-con-dengue-en-el-peru
